# Single-Trial EEG Connectivity of Default Mode Network Before and During Encoding Predicts Subsequent Memory Outcome

**DOI:** 10.3389/fnsys.2020.591675

**Published:** 2020-11-19

**Authors:** Dahye Kim, Woorim Jeong, June Sic Kim, Chun Kee Chung

**Affiliations:** ^1^Department of Brain and Cognitive Sciences, College of Natural Sciences, Seoul National University, Seoul, South Korea; ^2^College of Sungsim General Education, Youngsan University, Yangsan, South Korea; ^3^The Research Institute of Basic Sciences, College of Natural Sciences, Seoul National University, Seoul, South Korea; ^4^Department of Neurosurgery, Seoul National University Hospital, Seoul, South Korea; ^5^Neuroscience Research Institute, College of Medicine, Seoul National University, Seoul, South Korea

**Keywords:** memory, EEG, subsequent memory effects, functional connectivity, default mode network

## Abstract

The successful memory process produces specific activity in the brain network. As the brain activity of the prestimulus and encoding phases has a crucial effect on subsequent memory outcomes (e.g., remembered or forgotten), previous studies have tried to predict the memory performance in this period. Conventional studies have used the spectral power or event-related potential of specific regions as the classification feature. However, as multiple brain regions work collaboratively to process memory, it could be a better option to use functional connectivity within the memory-related brain network to predict subsequent memory performance. In this study, we acquired the EEG signals while performing an associative memory task that remembers scene–word pairs. For the connectivity analysis, we estimated the cross–mutual information within the default mode network with the time–frequency spectra at the prestimulus and encoding phases. Then, we predicted the success or failure of subsequent memory outcome with the connectivity features. We found that the classifier with support vector machine achieved the highest classification accuracy of 80.83% ± 12.65% (mean ± standard deviation) using the beta (13–30 Hz) connectivity at encoding phase among the multiple frequency bands and task phases. Using the prestimulus beta connectivity, the classification accuracy of 72.45% ± 12.52% is also achieved. Among the features, the connectivity related to the dorsomedial prefrontal cortex was found to contribute to successful memory encoding. The connectivity related to the posterior cingulate cortex was found to contribute to the failure of memory encoding. The present study showed for the first time the successful prediction with high accuracy of subsequent memory outcome using single-trial functional connectivity.

## Introduction

Differences in brain activity between the subsequently remembered and forgotten trials at learning are often referred to as subsequent memory effects (SMEs) (Paller and Wagner, 2002; Klimesch, 2012). As the brain activity during encoding has a crucial effect on subsequent memory outcomes, it is plausible to use it to predict the success of subsequent memory outcomes. A number of studies have successfully established that brain oscillations in multiple EEG frequency bands during the encoding phase can predict subsequently remembered and forgotten trials (Hanslmayr and Staudigl, 2014). In addition to SMEs during the encoding phase, previous studies also showed that prestimulus activities could predict subsequent memory outcome, as anticipatory activity prior to stimulus presentation could play a critical role in how a stimulus will be processed (Otten et al., 2006; Guderian et al., 2009; Addante et al., 2011, 2015; Burke et al., 2014; Cohen et al., 2015; Schneider and Rose, 2016).

In this regard, there have been studies that predicted subsequent memory outcome (e.g., remembered or forgotten) using single-trial EEG SMEs features of prestimulus, and during the encoding phase. By combining the prestimulus and during encoding phase SMEs, a previous study achieved 59.6% classification accuracy (Noh et al., 2014), whereas another study achieved 72.1% classification accuracy (Sun et al., 2016). These studies have used the spectral power or event-related potential (ERP) of specific regions to predict subsequent memory outcome. However, as the brain works collaboratively to process memory, other than features of local signal amplitude, a better option to predict subsequent memory outcome could be to use the functional connectivity between multiple brain regions.

The activities of the medial temporal lobe are well-known to be related to successful memory encoding (Guderian et al., 2009; Lega et al., 2012). In addition, widespread brain area, especially belonging to the default mode network (DMN), which is a common set of brain regions that demonstrate consistently greater activity while resting than while performing cognitively demanding tasks, such as memory task (Buckner et al., 2008), is also known to be related to successful memory function (Kim, 2011). This network includes the medial prefrontal cortex, the posterior parietal cortex, the hippocampus (HC), the precuneus, the inferior parietal lobe, and the lateral temporal lobe. The deactivation of the DMN during encoding is known to reflect successful memory encoding (Anticevic et al., 2010; Chai et al., 2014; Raichle, 2015; Sato and Mizuhara, 2018); meanwhile, the activation of the DMN during encoding is known to be related to subsequent forgetting (Kim, 2011). Specifically, in a previous study, distinct DMN regions were reported to modulate both encoding success and failure. The medial prefrontal cortex, angular gyrus (AG), and lateral temporal cortex were reported to mediate encoding success in a self-referential memory encoding task, whereas the posterior cingulate was reported to be related to both encoding failure and task-unrelated thought (Maillet and Rajah, 2014). In addition, when individuals constructed mental scenes based on memory, a distinct subsystem of the DMN was preferentially engaged (Andrews-Hanna et al., 2010). Additionally, the posterior cingulate, inferior parietal cortex, and lateral temporal cortex were recruited during the construction of the mental scenes, which is the process that could be required in the memory encoding (Hassabis et al., 2007). In summary, previous studies have shown that various brain areas involved in the DMN have an important role in the success and failure of the memory encoding process.

Despite the importance of DMN activity, no studies to date have predicted subsequent memory outcome using the single-trial functional connectivity of DMN regions in prestimulus and during the encoding phase. Here, we hypothesized that connectivity features could predict subsequent memory outcome in single-trials. Therefore, we predicted subsequent memory outcome using features from the frequency specific connectivity within DMN. Here, among the various connectivity measures, we used mutual information (MI) that evaluates the amount of information about one signal that is contained in another signal (Fraser and Swinney, 1986).

## Materials and Methods

### Participants

Twenty-nine right-handed healthy subjects without neurological or psychiatric abnormalities were recruited. The datasets of two participants were excluded from further analysis because of insufficient artifact-free trials. The final study group included 27 participants (11 females; mean age = 26.0 ± 2.1 years, range = 23–31 years; education level = 17.0 ± 1.2 years). As an effort to control the physiological condition of subjects, we instructed the subjects to get enough sleep the day before they participated in the experiment. This study was approved by the institutional review board (IRB) of the Seoul National University Hospital Clinical Research Institute (IRB number: H-1808-098-967).

### Experimental Paradigm

**FIGURE 1:**
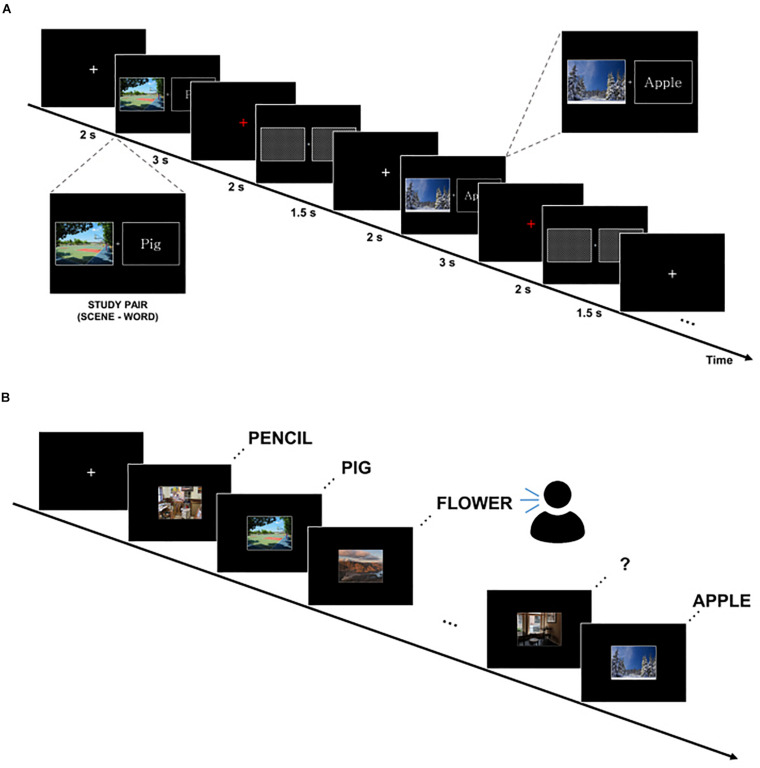
Experimental paradigm. **(A)** Study block. Subjects were instructed to remember scene–word pairs using a strategy that makes an imaginary associative scene. **(B)** Test block. Subjects were asked to verbally recall the word cued by the scene at self-paced speed.

We applied a subsequent memory paradigm that consisted of scene–word pairs in study and test blocks (Figure 1). Each study and test block had 75 trials. Scene stimuli consisted of 50% indoor images and 50% outdoor images. As word stimuli, concrete nouns of 50% nature objects and 50% manmade objects were used. During the study phase, subjects were instructed to remember scene–word pairs using a strategy that made an imaginary scene associated with the paired word for 3 s. In order to boost subjects’ participation in the experiment, the subjects were instructed to press a button on whether or not the associative imaginary scene in the mind vividly appeared when a red fixation was presented on the screen. To eliminate the residual effect of the previous stimulus, boxes filled diagonally were presented for 1.5 s. The encoding phase of one experimental session lasted about 10 min. After the study block, subjects were instructed to answer as many simple arithmetic problems as possible in 2 min for a distracting task (e.g., alternately subtract 4 and 7 from 100). During the test phase, subjects were instructed to verbally recall the word cued by the scene previously learned at self-paced speed. There was a time limit of up to 20 s, and if exceeded, it was classified as forgotten. To maintain the attention of the participants, the next session went on when the subjects were fully prepared. One session consisted of a block of study, distraction, and test. Three sessions were conducted for each subject. Stimuli were presented using STIM2 presentation software (Compumedics Neuroscan, Australia).

### EEG Data Acquisition and Preprocessing

EEG was recorded using a Neuroscan EEG recording system (Compumedics Neuroscan, United States) with 64-channel Quick caps (Compumedics Neuroscan, United States) in an electrically shielded cabin. EEG signals were digitized at a sampling frequency of 1,000 Hz with a SynAmps2 amplifier (Compumedics Neuroscan, United States). Preprocessing was conducted using EEGLAB open source toolbox (version 2019.1^1^) and self-written MATLAB scripts (version R2019b; MathWorks Corp.). At the first preprocessing step, the linked ear EEG montage was converted to common average reference. The data were segmented into epochs ranging from -1,000 to 2,000 ms relative to the stimulus onset. Trials that include high noise were manually discarded, and only the remaining artifact-free trials were used for further classification analysis. Table 3 shows the final included number of trials for each individual.

### Frequency-Specific EEG Source Connectivity Analysis

For the time–frequency analysis, continuous wavelet transformation was applied. We focused on the theta (3–7 Hz), alpha (8–12 Hz), and beta (13–30 Hz) frequency bands. These power values were then normalized by the baseline activity before the stimulus onset of −1 to −0.8 s. EEG signals at -500 to 0 ms before the stimulus onset were used as a “prestimulus” phase, and EEG signals after the stimulus onset at 0 to 1,000 ms were used as an “encoding” phase for subsequent connectivity analysis. To take into account the time to see and recognize the stimuli, the encoding phase was designated as 1,000 ms. For the source analysis, regions of interest (ROIs) were selected in regions that were representative of the DMN (Raichle, 2015). The ROIs included the dorsomedial prefrontal cortex (DMPFC), posterior cingulate cortex (PCC), AG, middle temporal gyrus (MTG), and HC. Table 1 shows the coordinates of the ROIs. Source activity was extracted using the built-in function of discrete model probing in BESA research 6.0 (GmbH, Germany).

**TABLE 1:** Talairach coordinates of regions of interest.

**Region**	**Abbreviation**	***x***	***y***	***z***
Dorsomedial prefrontal cortex	DMPFC	0	32	5
Posterior cingulate cortex	PCC	0	−51	23
Left angular gyrus	AG (L)	−44	−54	23
Right angular gyrus	AG (R)	44	−54	23
Left middle temporal gyrus	MTG (L)	−53	−2	−18
Right middle temporal gyrus	MTG (R)	53	−2	−18
Left hippocampus	HC (L)	−27	−11	−13
Right hippocampus	HC (R)	27	−11	−13

For the source connectivity analysis, we calculated the time–frequency cross-MI (Jeong et al., 2001). After the continuous wavelet transform, the mean value of each frequency band (theta, alpha, and beta) was obtained. Then, cross-MI was calculated using samples from each time interval (prestimulus and encoding phase). MI is a measure of the amount of dependency between two signals. Compared to linear correlation, MI is a more general measurement, because it can measure non-linear dependency. The temporal series of averaged frequency band signals were used to compute the cross-MI between ROIs. MI values between ROIs can be calculated using the probability density function, as follows:

$$M\,I=M\,I_{X\,Y}=M\,I_{Y\,X}=M\,I\,\left(X,Y\right)=$$

$$\sum p\,\left(X,Y\right)\,\log\frac{p\,\left(X,Y\right)}{p\,\left(X\right)\cdot p\,\left(Y\right)}$$

here, *p(X, Y)* is the joint probability distribution function of variables *X* and *Y*, and *p(X)* and *p(Y)* are the marginal probability distribution functions of *X* and *Y*, respectively.

Then, we investigated the differences in EEG connectivity between subsequently remembered and forgotten trials (during both the Prestimulus and Encoding phases).

### Two-Class Classification Using Single-Trial Source Connectivity Features

**FIGURE 2:**
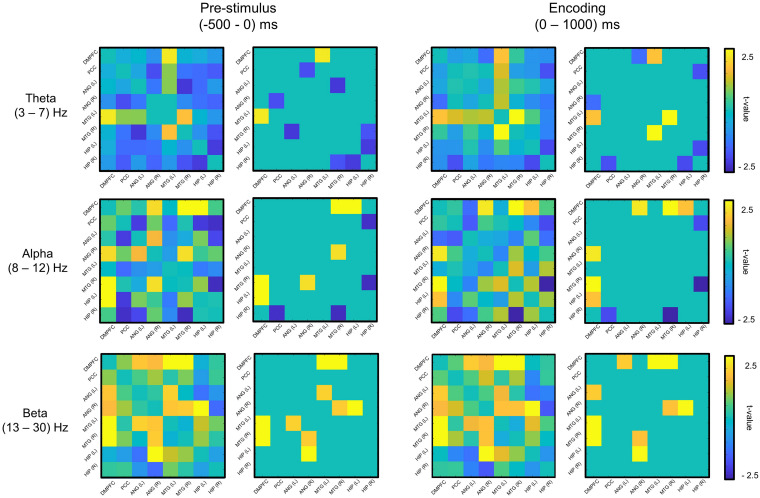
Feature selection of mutual information (remembered–forgotten). For the feature selection, as a result of the *t*-test for the difference between the remembered and forgotten conditions (left column of each phase), features with the absolute value of the *t*-value in the top 20% (right column of each phase) were selected in each phase (e.g., prestimulus and encoding phases). DMPFC, dorsomedial prefrontal cortex; PCC, posterior cingulate cortex; ANG, angular gyrus; MTG, middle temporal gyrus; HIP, hippocampus; L, left; R, right.

We used a linear support vector machine (SVM) in MATLAB for the classification of memory success. The most informative connectivity values with the top 20% of the *t*-statistics were selected as the features in each phase (i.e., the prestimulus and encoding phase) and each frequency band (Figure 2). The classification performance of individual EEG signal was evaluated by fivefold cross-validation with 100 repetitions.

## Results

### Behavioral Results

On average, participants correctly remembered 54.64% ± 18.26% (mean ± standard deviation) trials of the stimulus, indicating that they were able to efficiently encode materials and that we obtained enough trials for both remembered and forgotten conditions.

### Feature Selection

Figure 2 shows the selected features for each phase and frequency band, and Table 2 presents the *t*-statistics values (uncorrected) and regions of the selected connectivity. The most informative connectivity values with the top 20% of the *t*-statistics (uncorrected) were selected as the features in each phase (i.e., the prestimulus and encoding phase) and frequency band. The subsequently remembered condition showed increased connectivity centered on the DMPFC at the prestimulus and the encoding phase. On the other hand, the subsequent forgotten condition showed increased connectivity centered on PCC in the theta and alpha bands at the prestimulus phase.

**TABLE 2:** Results of the *t*-test for the difference between the remembered and forgotten conditions.

**Time**	**Band**	**Feature set**		***t*-value**	**Time**	**Band**	**Feature set**		***t*-value**
Prestimulus	Theta	DMPFC	MTG (L)	2.282*	Encoding	Theta	MTG (L)	MTG (R)	2.891**
		MTG (L)	MTG (R)	1.734*			DMPFC	MTG (L)	1.776*
		MTG (R)	HIP (R)	−1.760*			PCC	MTG (L)	1.367
		PCC	ANG (R)	−1.848*			DMPFC	ANG (R)	−1.393*
		HIP (L)	HIP (R)	−2.096*			HIP (L)	HIP (R)	−1.890*
		ANG (L)	MTG (R)	−2.139*					
	Alpha	DMPFC	HIP (L)	2.941**		Alpha	DMPFC	MTG (R)	3.324**
		DMPFC	MTG (R)	2.818**			DMPFC	ANG (R)	2.256*
		DMPFC	ANG (R)	2.077*			HIP (L)	HIP (R)	1.759*
		ANG (L)	HIP (R)	−1.922*			PCC	HIP (R)	−1.879*
		PCC	HIP (L)	−2.119*			MTG (R)	HIP (R)	−2.728*
		PCC	HIP (R)	−2.271*					
		MTG (R)	HIP (R)	−2.334*					
	Beta	DMPFC	MTG (L)	3.810**		Beta	DMPFC	MTG(L)	3.661**
		DMPFC	MTG (R)	2.680**			DMPFC	MTG(R)	2.859**
		ANG (R)	HIP (L)	2.625**			ANG(R)	HIP(L)	2.475*
		ANG (L)	MTG (L)	1.958*			DMPFC	ANG(L)	1.878*
		ANG (R)	MTG (R)	1.880*			ANG(R)	MTG(R)	1.763*
		ANG (R)	MTG (L)	1.831*			ANG(R)	MTG(L)	1.748*
		DMPFC	ANG (L)	1.829*					

*p < 0.05, **p < 0.01.DMPFC, dorsomedial prefrontal cortex; PCC, posterior cingulate cortex; ANG, angular gyrus; MTG, middle temporal gyrus; HIP, hippocampus; L, left; R, right.

### Classification Accuracy

Table 3 describes the individual classification accuracy in each phase and frequency band. The SVM binary classifier achieved the highest mean classification accuracy of 80.83% ± 12.65% (mean ± standard deviation) using the beta connectivity features at the encoding phase (chance level = 50%). At the encoding phase, an accuracy of 72.79% ± 12.85% was achieved using the alpha connectivity features, and 69.36% ± 11.90% was achieved using the theta connectivity features. Using the prestimulus beta connectivity features, the mean classification accuracy was also 72.45% ± 12.52%. At the prestimulus phase, an accuracy of 66.92% ± 11.93% was achieved using the alpha connectivity features, and 61.15% ± 10.91% was achieved using the theta connectivity features.

**TABLE 3:** Individual classification accuracy.

	**Prestimulus (−500 to 0 ms) (%)**			**Encoding (0–1,000 ms) (%)**			**No. of trials (REM/FOR)**
**Subject**	**Theta**	**Alpha**	**Beta**	**Theta**	**Alpha**	**Beta**	
sub1	53.83	79.95	56.93	61.53	74.38	72.93	84/101
sub2	66.00	82.97	75.83	66.39	90.44	91.58	108/72
sub3	52.50	56.71	93.66	73.63	69.16	87.03	146/40
sub4	60.76	94.13	73.45	59.63	96.16	57.95	107/83
sub5	56.36	75.24	83.12	56.14	71.19	88.57	109/101
sub6	71.17	64.40	76.95	60.86	85.90	60.55	97/99
sub7	52.98	53.03	78.40	56.60	53.38	91.78	58/132
sub8	70.16	64.09	67.31	77.20	73.43	72.41	127/89
sub9	60.88	59.83	67.53	77.13	81.80	68.30	115/80
sub10	55.78	79.06	85.56	67.69	81.75	92.16	81/78
sub11	50.60	74.02	83.50	78.07	83.21	92.55	144/64
sub12	57.42	70.68	91.32	81.87	74.45	89.34	120/71
sub13	52.95	61.21	93.76	73.05	52.37	99.39	84/101
sub14	60.66	66.07	86.07	68.30	75.32	93.80	150/66
sub15	51.94	57.00	56.42	55.58	64.25	73.89	28/148
sub16	57.00	56.3	64.75	75.16	56.61	74.41	172/47
sub17	50.82	64.39	53.42	56.37	61.13	55.71	169/30
sub18	59.21	69.75	69.17	75.25	63.50	75.17	70/52
sub19	85.03	77.00	85.38	82.75	68.44	88.06	101/61
sub20	54.97	54.00	66.50	58.82	77.47	65.97	117/51
sub21	55.11	53.34	63.29	69.08	65.08	85.13	142/43
sub22	52.98	63.38	48.21	46.21	66.95	67.12	92/117
sub23	53.55	50.14	64.66	58.25	52.11	77.89	31/190
sub24	76.11	61.24	67.74	92.18	81.24	84.74	100/92
sub25	62.45	51.74	72.32	67.18	59.84	84.08	58/132
sub26	77.40	78.15	73.03	83.68	91.35	92.30	73/130
sub27	92.42	89.08	57.79	94.03	94.42	99.67	74/109
Average	61.15	66.92	72.45	69.36	72.79	80.83	
SD	10.91	11.93	12.52	11.90	12.85	12.65	

## Discussion

The present study showed that subsequent memory outcome (e.g., remembered or forgotten) can be successfully predicted using the functional connectivity within DMN regions.

### Subsequent Memory Effects of Functional Connectivity

In the present study, using prestimulus beta band connectivity, we achieved 72.45% average classification accuracy, and using encoding beta band connectivity, we achieved 80.83% prediction accuracy. By using functional connectivity features, we achieved higher classification accuracy than the previous studies that used local brain activities.

Conventional studies have investigated the role of local brain activities in relation to the formation of human memories. Previous studies have consistently reported that successful memory encoding is related to the activation of specific brain regions. In particular, successful memory encoding is related to the medial temporal lobe and prefrontal cortex (Wagner et al., 1998; Paller and Wagner, 2002; Reber et al., 2002; Kim, 2011), whereas the failure of memory encoding is related to PCC and temporoparietal junction (Otten and Rugg, 2001; Kim, 2011). In addition to encoding SMEs, as anticipatory activity prior to stimulus presentation could play a critical role in how a stimulus will be processed, many studies have also investigated prestimulus SMEs (Otten et al., 2006; Guderian et al., 2009; Addante et al., 2011, 2015; Burke et al., 2014; Cohen et al., 2015; Schneider and Rose, 2016). However, recent studies have suggested the involvement of the more widely distributed cortical network and the importance of its collaborative roles in the episodic encoding (Jeong et al., 2015).

Previous studies have predicted subsequent memory outcome based on regional activity. By combining prestimulus and encoding SMEs, one study achieved 59.6% classification accuracy (Noh et al., 2014), whereas another study achieved 72.1% (Sun et al., 2016). They used brain activity in specific areas (e.g., spectral power or ERP of specific regions) as a feature. On the other hand, our results showed that subsequent memory outcome could be better predicted using functional connectivity compared to the local brain activity. Our approach would reflect the way in which the brain regions related to a memory task work together.

### Characteristics of a Successful Memory Network at the Prestimulus and Encoding Phases

At the prestimulus phase, to prepare for a successful encoding, it is necessary to maintain top–down attention to task and to suppress task-irrelevant thoughts that occur before the stimulus onset. Therefore, ongoing neural activity occurring before the stimulus onset might play an important role in preparing the brain for successful memory outcome (Otten et al., 2006; Guderian et al., 2009; Addante et al., 2011, 2015; Burke et al., 2014; Cohen et al., 2015; Schneider and Rose, 2016). In our results, the connectivity centered on the DMPFC at the prestimulus phase was related to the subsequently remembered condition. The connectivity of the DMPFC was reported to be related to maintaining top–down attention and focus on task for successful encoding (Miller, 2000).

At the encoding phase, the process of focusing on the task and associating scene and word is required for successful encoding. In our results, the connectivity centered on the DMPFC was maintained, and theta band connectivity between the bilateral MTG and alpha band connectivity between the DMPFC and MTG (R) was increased, compared to the prestimulus phase. The maintained connectivity centered on the DMPFC may reflect top–down attention to the task. For the increased connectivity centered on the MTG, as the MTG is known to be involved in semantic processing (Vandenberghe et al., 1996; Visser et al., 2012), associating scene and word for successful memory formation might reflect semantic processing.

### Dissociable Roles of DMN Regions During the Prestimulus and Encoding Phases

**FIGURE 3:**
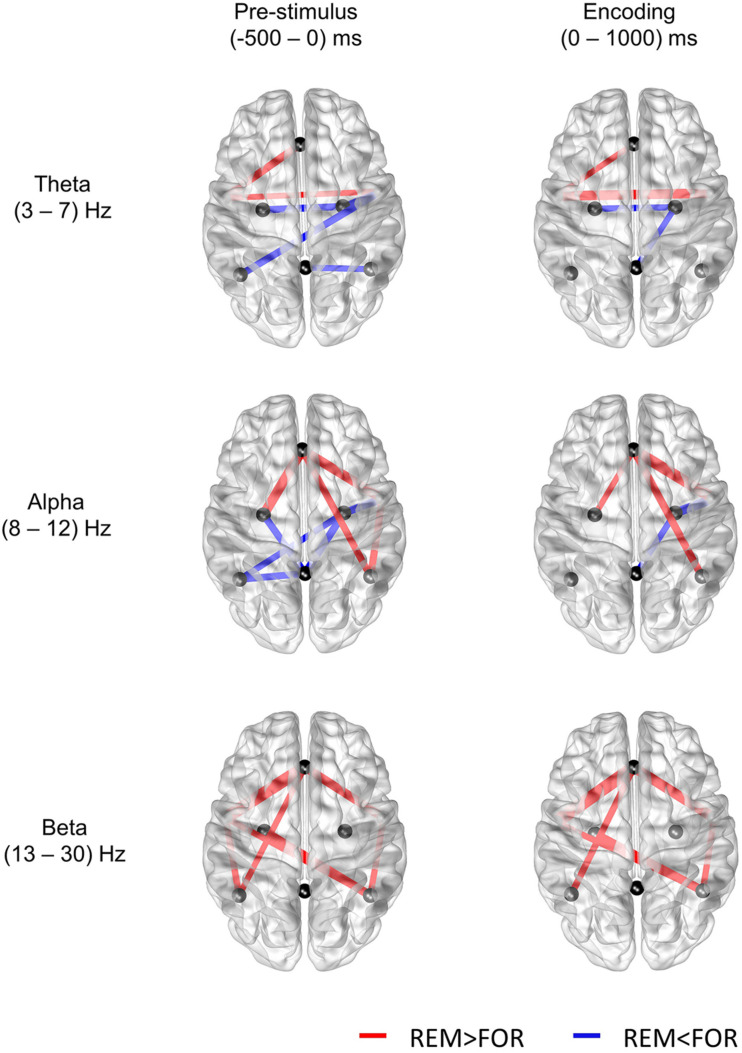
Results of the *t*-test for the difference in mutual information between the remembered and forgotten conditions (*p* < 0.05). The nodes selected in this study are the midline DMPFC, midline PCC, left and right ANG, right and left MTG, and right and left HIP.

Previously, distinct DMN regions have been reported to modulate both encoding success and failure. Medial prefrontal cortex and AG were reported to mediate encoding success, whereas PCC was reported to be related to both encoding failure and task-unrelated thought (Maillet and Rajah, 2014). Similarly, the PCC showed more activity in subsequent forgotten items than the subsequently remembered items (Otten and Rugg, 2001; Wagner and Davachi, 2001; Daselaar et al., 2004, 2009). In our results, the connectivity centered on the DMPFC was found to be related to successful memory encoding, whereas the connectivity centered on the PCC was found to be related to the failure of memory encoding. In particular, the higher connectivity centered on the PCC in the forgotten conditions was obvious in the prestimulus phase. To sum up, successful memory encoding was found to be related to the connectivity of the anterior DMN, whereas the failure of memory encoding was found to be related to the connectivity of the posterior DMN (Figure 3).

## Conclusion

This is the first study in which subsequent memory outcome has been predicted using single-trial functional connectivity in the prestimulus and encoding phases using scalp EEG. In this study, using single-trial EEG connectivity features, we achieved average accuracy of greater than 80% for the prediction of subsequent memory outcome. We found anterior and posterior dissociation of the DMN. In both the prestimulus and encoding phases, in remembered conditions, the connectivity of the anterior DMN was higher, whereas that of the posterior DMN was lower. In the future, these results could be very useful in building a closed-loop brain stimulation system for memory enhancement that could deliver stimulation only when a subsequent memory outcome is predicted to be forgotten (Ezzyat et al., 2018).

## Data Availability Statement

The raw data supporting the conclusions of this article will be made available by the authors, without undue reservation.

## Ethics Statement

The studies involving human participants were reviewed and approved by the Seoul National University Hospital Clinical Research Institute (IRB number: H-1808-098-967). The participants provided their written informed consent to participate in this study.

## Author Contributions

DK, WJ, JK, and CC contributed to the study design and wrote the manuscript. DK and WJ performed the study. DK and JK analyzed the data. JK and CC obtained funding. All authors contributed to the article and approved the submitted version.

## Conflict of Interest

The authors declare that the research was conducted in the absence of any commercial or financial relationships that could be construed as a potential conflict of interest.

## Footnotes

1 http://sccn.ucsd.edu/eeglab
